# CTBPro: A Next‐Generation Cholera Toxin Subunit B‐Based Neuroanatomical Tracer With Superior Brightness, Stability, and Sensitivity for Enhanced Neural Circuit Mapping

**DOI:** 10.1002/advs.202522249

**Published:** 2026-04-22

**Authors:** Xinghua Quan, Yude Lou, Huiqi Xie, Yifei Wang, Yanzhe Zhang, Yiran Ge, Linhe Yang, Xiaoxuan Zhang, Qingmiao Zhou, Huaizong Shen, Longxing Cao, Xin Jin, Jie‐Min Jia

**Affiliations:** ^1^ Laboratory of Neurovascular Biology, School of Life Sciences Westlake University Hangzhou Zhejiang China; ^2^ Westlake Laboratory of Life Sciences and Biomedicine Hangzhou Zhejiang China; ^3^ Zhejiang Key Laboratory of Structural Biology, School of Life Sciences Westlake University Hangzhou Zhejiang China; ^4^ State Key Laboratory of Gene Expression, School of Life Sciences Westlake University Hangzhou Zhejiang China; ^5^ School of Life Sciences Westlake University Hangzhou Zhejiang China; ^6^ Institute of Basic Medical Sciences Westlake Institute for Advanced Study Hangzhou Zhejiang China

**Keywords:** cholera toxin subunit b, high‐resolution neural imaging, protein‐based fluorescent tracer, retrograde tracing

## Abstract

Mapping complex neural circuits demands bright and stable tracers, yet conventional Cholera Toxin Subunit B (CTB) conjugates exhibit suboptimal brightness, which limits high‐fidelity morphological reconstruction. Here, we engineer CTBPro, a next‐generation CTB‐based tracer, by genetically fusing CTB to the ultra‐stable fluorescent protein mBaojin. In vitro, CTBPro exhibits a calculated nine‐fold enhancement in molar brightness compared to conventional CTB‐Alexa conjugates. This enhanced brightness translates to superior in vivo tracing across four distinct administration routes—intraparenchymal, peripheral (renal), cerebrospinal fluid (CSF), and intravenous—enabling high‐fidelity labeling of fine somatic and axonal details. Critically, because CTBPro is an entirely protein‐based fusion, it can be genetically encoded. We leverage this property by packaging CTBPro into an AAV‐BI30 serotype vector, which enables endothelial‐specific expression that recapitulates the distinctive labeling pattern of intravenous injection. Taken together, CTBPro overcomes the fundamental limitations of CTB‐based tracers. Its superior brightness, versatile administration, and multimodal stability establish it as a powerful and extensible tool for high‐resolution neuronal circuit tracing.

## Graphical abstract

CTBPro is a next‐generation cholera toxin B–based tracer engineered by fusing CTB to the ultra‐stable fluorescent protein mBaojin. Exhibiting markedly enhanced molar brightness, CTBPro enables high‐fidelity neuronal labeling across multiple administration routes. Its genetically encodable design and AAV compatibility further expand its versatility, establishing CTBPro as a powerful tool for high‐resolution neural circuit mapping.

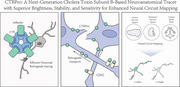

## 1 Introduction

Mapping neural circuits is a fundamental pursuit in neuroscience, offering essential insights into the complex connectivity and functional organization of the nervous system [1]. Over the past decades, the development of neural tracing methodologies has significantly advanced our ability to interrogate these circuits. Early approaches utilized proteins such as horseradish peroxidase (HRP) and wheat germ agglutinin (WGA) to map neuronal pathways [2, 3]. However, these tracers were constrained by inherent limitations, such as their inability to reliably determine signal directionality and complications from multi‐synaptic tracing [3, 4, 5, 6].

Subsequent innovations introduced viral tracers, including adeno‐associated virus retrograde serotype (AAV‐retro) and rabies virus (RV), which enhanced circuit mapping by enabling trans‐synaptic propagation [7, 8]. However, the application of viral tracers is frequently compromised by substantial neurotoxicity, making functional studies difficult [9, 10, 11, 12, 13].

The introduction of cholera toxin subunit B (CTB) as a retrograde tracer marked an advancement in neural circuit mapping, enabling directional, non‐trans‐synaptic tracing [14, 15]. Enhancements such as CTB conjugated to colloidal gold nanoparticles (CTB‐gold) improved stability and compatibility with immunohistochemistry but required complex silver enhancement staining, limiting practical use [16, 17]. Chemical fluorophore conjugates such as CTB‐Alexa488 offered greater convenience through direct fluorescence visualization [18, 19]. However, these conventional tracers still face critical limitations, including low fluorescence brightness, inadequate morphological detail, incompatibility with advanced techniques such as tissue clearing, and the inability to be genetically expressed in vivo, all of which restrict their broader applicability.

To overcome these challenges, we developed CTBPro, a novel tracer created by fusing CTB with mBaojin, a green fluorescent protein distinguished by its exceptional brightness and photostability [20]. CTBPro exhibits high fluorescence intensity and is compatible with diverse experimental modalities. It enables precise retrograde tracing in both the central and peripheral nervous systems, allowing for the visualization of neuronal morphology with high clarity and detail. Furthermore, as CTBPro is protein‐based, it can be endogenously expressed using viral vectors. This design as a genetic fusion establishes CTBPro as an extensible platform, allowing it to be readily fused with other advanced fluorescent proteins (e.g., myonghong, emiRFP670) to create a multi‐color tracing palette [21, 22]. This adaptability expands its versatility for complex, multi‐channel neuroscience research.

## 2 Results

### 2.1 Design and Characterization of CTBPro

**FIGURE 1:**
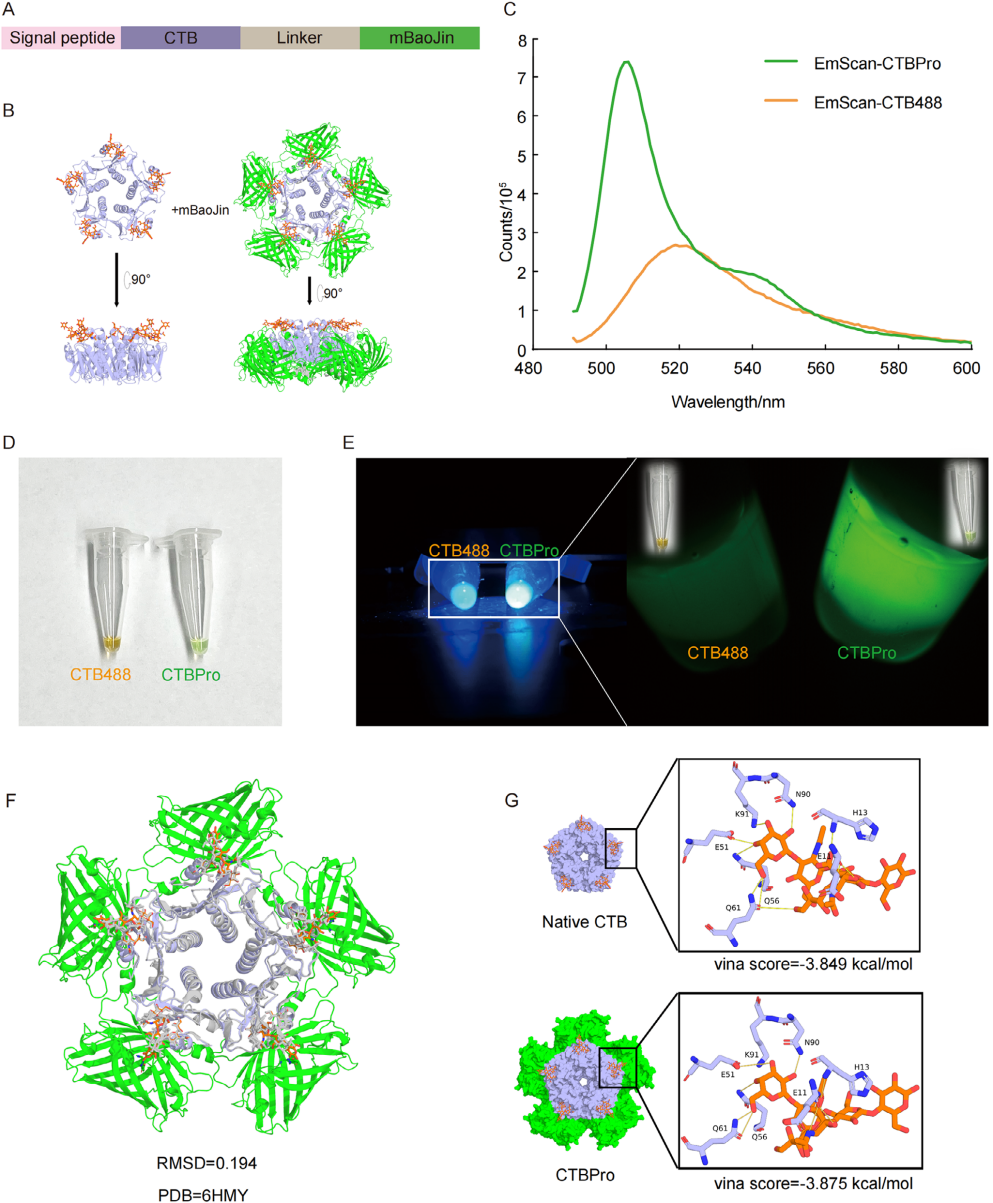
Design, structural characterization, and in silico analysis of CTBPro. (A) Schematic of the CTBPro fusion protein design. (B) Structural details of native CTB and the predicted CTBPro fusion. (C) Comparison of fluorescence intensity between CTBPro and CTB‐Alexa488 at various excitation wavelengths, measured by fluorescence spectroscopy. (D) Bright‐field images of CTB‐Alexa488 and CTBPro solutions. (E) Comparison of fluorescence brightness between CTB‐Alexa488 and CTBPro in the 488 nm channel. (F) Structural alignment and comparison between predicted CTBPro (light purple) and native CTB (light gray) (PDB: 6HMY). (G) Predicted docking poses of the GM1 ganglioside with native CTB and CTBPro.

To enhance the utility of CTB as a neural tracer, we developed CTBPro. This genetically engineered construct fuses CTB to mBaojin, a highly photostable green fluorescent protein, via a flexible linker. To optimize expression, we added an N‐terminal signal peptide and performed mammalian codon optimization (Figure 1A). CTBPro was successfully expressed in 293F cells and purified to homogeneity. Biochemical characterization revealed that the purified protein migrates at an apparent molecular weight of ∼48 kDa on SDS‐PAGE due to extensive post‐translational glycosylation, as confirmed by the mobility shift following enzymatic deglycosylation (Figure S1A). High‐resolution intact mass spectrometry and peptide mapping of the deglycosylated sample validated the protein identity with ≈97% sequence coverage and a precise mass match (Figure S1B–D, Tables S1, S2).

At 2.5 µg/µL (the concentration commonly used for commercial CTB‐Alexa488), CTBPro showed a stronger green signal under bright‐field microscopy. Fluorescence microscopy further revealed that CTBPro had significantly higher fluorescence intensity in the 488 nm excitation channel than CTB‐Alexa488 (Figure 1D,E). Notably, despite the same mass concentration (2.5 µg/µL), the molecular weight of CTBPro is approximately threefold higher than that of CTB‐Alexa488. Thus, on a molar basis, CTBPro achieved superior fluorescence at only one‐third the molar concentration. For consistency, all subsequent experiments used the same 2.5 µg/µL mass concentration for both tracers.

Quantitative photoluminescence spectrometry further validated the enhanced performance of CTBPro, showing fluorescence intensity greater than CTB‐Alexa488, with an increase of nearly 3‐fold (Figure 1C, Table S3). Beyond its superior optical properties, we investigated whether the structural integrity of CTBPro could withstand harsh chemical environments. Solvent‐based tissue clearing protocols (e.g., iDISCO) rely on aggressive dehydration and delipidation steps that often quench conventional fluorophores. Remarkably, we found that CTBPro retained detectable fluorescence throughout this process (Figure S2). This chemical resilience, combined with its high brightness, highlights the robust stability of the mBaojin fusion protein.

To investigate whether the fusion design affected the structural or functional properties of CTB, we performed structural prediction using AlphaFold3. Comparative analyses between the predicted CTBPro structure and the known crystal structure of CTB indicated no discernible alterations in the conformation of the CTB domain (Figure 1F). Additionally, molecular docking assays confirmed that CTBPro retained the ability to bind GM1 gangliosides, a critical functional property of CTB, with no observable differences from native CTB (Figure 1G). To further evaluate our predictions, we assessed the binding of CTBPro to GM3. The docking results demonstrated that the affinity between GM3 and CTBPro was lower than that for GM1, consistent with expectations (Figure S3).

### 2.2 Retrograde Tracing With CTBPro in the Central Nervous System

**FIGURE 2:**
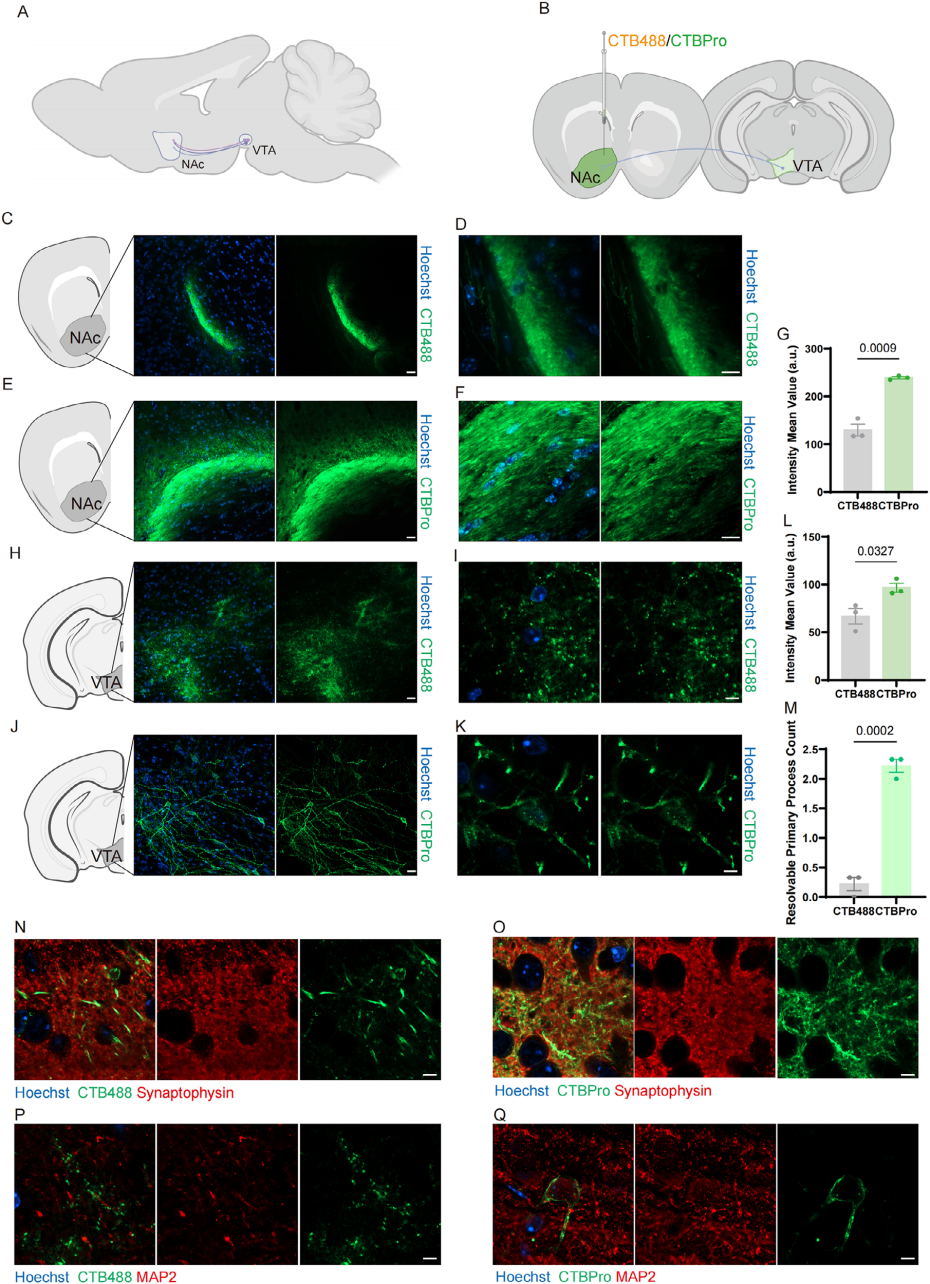
**CTBPro enables enhanced in vivo retrograde tracing of the VTA‐NAc circuit**. (A) Schematic illustration of the ventral tegmental area (VTA) to nucleus accumbens (NAc) circuit. (B) Schematic diagram of CTBPro or CTB‐Alexa488 stereotactic injection into the NAc. (C) Representative image of the NAc region 7 days post‐injection with CTB‐Alexa488. Confocal: 20X. Scale bar: 20 µm. Laser: 488 nm, 0.2%. (D) Representative image of the NAc region 7 days post‐injection with CTB‐Alexa488. Confocal: 63X. Scale bar: 10 µm. Laser: 488 nm, 0.5%. (E) Representative image of the NAc region 7 days post‐injection with CTBPro. Confocal: 20X. Scale bar: 20 µm. Laser: 488 nm, 0.2%. (F) Representative image of the NAc region 7 days post‐injection with CTBPro. Confocal: 63X. Scale bar: 10 µm. Laser: 488 nm, 0.2%. (G) Quantification of fluorescence intensity at the NAc injection site for CTB‐Alexa488 and CTBPro. (H) Representative image of the VTA region 7 days post‐injection with CTB‐Alexa488. Confocal: 20X. Scale bar: 20 µm. Laser: 488 nm, 1%. (I) Representative image of the VTA region 7 days post‐injection with CTB‐Alexa488. Confocal: 63X. Scale bar: 5 µm. Laser: 488 nm, 1%. (J) Representative image of the VTA region 7 days post‐injection with CTBPro. Confocal: 20X. Scale bar: 20 µm. Laser: 488 nm, 1%. (K) Representative image of the VTA region 7 days post‐injection with CTBPro. Confocal: 63X. Scale bar: 5 µm. Laser: 488 nm, 1%. (L) Quantification of fluorescence intensity of retrograde‐labeled soma in the VTA. (M) Quantification of resolvable primary processes per soma in the VTA. (N‐O) Colocalization of axonal signals and synaptophysin near the injection site. (P‐Q) Colocalization of somatic signals and MAP2 in the retrograde‐labeled target region (VTA). Each data point represents one mouse (N = 3 per group). Statistical significance was assessed using Student's t‐test.

To validate the in vivo neuronal tracing capabilities of CTBPro, we investigated its performance in a well‐characterized central nervous system circuit: the projection from the ventral tegmental area (VTA) to the nucleus accumbens (NAc) (Figure 2A,B). Specifically, 100 nL of CTB‐Alexa488 or CTBPro was injected into the NAc of mice. All brain samples were harvested for imaging 7 days post‐injection.

Imaging results demonstrated that CTBPro effectively labeled axons within the NAc region, producing clear and robust signals even at a relatively low confocal laser intensity (Figure 2E,F). Notably, CTBPro facilitated detailed neuronal labeling, enabling the visualization of distinct neuronal cell bodies and comprehensive morphological information, including neuronal soma in the VTA (Figure 2J,K).

We compared these results to commercially available CTB‐Alexa488, applied using identical parameters. While CTB‐Alexa488 successfully traced neuronal projections, its imaging quality under identical laser intensity was significantly inferior to that of CTBPro. Specifically, CTB‐Alexa488 failed to achieve comparable resolution in neuronal morphology and struggled to capture detailed dendritic and axonal processes (Figure 2C,D,H,I).

To evaluate whether CTBPro labeled neuron axons, we used synaptophysin as a marker and found that CTBPro showed better colocalization with synaptophysin (Figure 2N,O). Similarly, in the target brain regions, immunostaining with the somatic marker MAP2 also suggested better colocalization (Figure 2P,Q). We further quantified these in vivo observations, confirming that CTBPro exhibited significantly higher fluorescence intensity (Figure 2G,L). Furthermore, a blinded count of resolvable primary processes demonstrated the morphological superiority: CTBPro‐labeled neurons revealed detailed processes, whereas CTB‐Alexa488‐labeled neurons, appearing only as diffuse signals, provided minimal morphological detail (Figure 2M).

### 2.3 Retrograde Tracing With CTBPro in the Peripheral Nervous System

**FIGURE 3:**
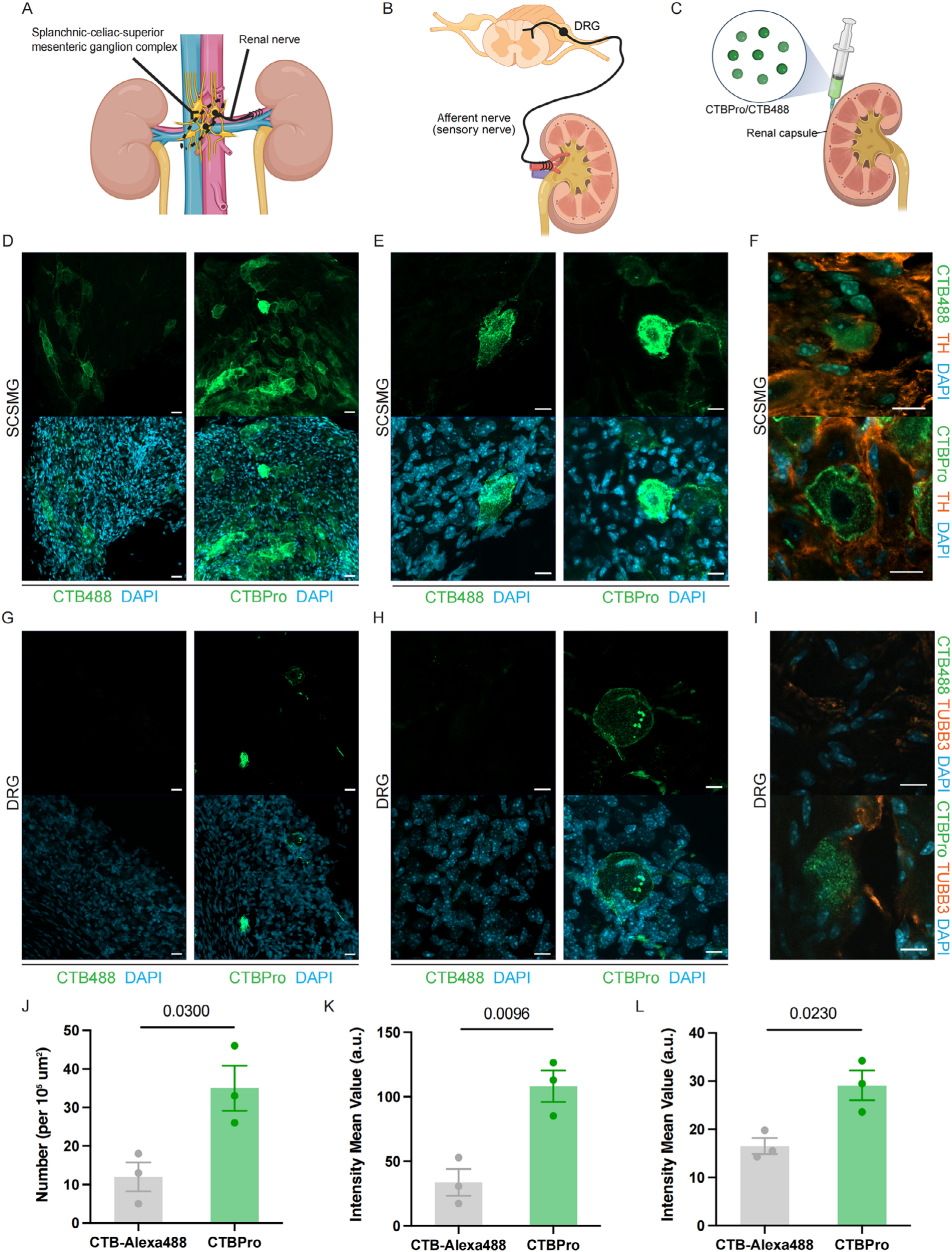
**CTBPro demonstrates superior renal neural tracing**. (A) Schematic illustration of efferent fibers (postganglionic sympathetic fibers) innervating the kidney originate from the splanchnic–celiac–superior mesenteric ganglion complex (SCSMG). (B) Schematic illustration of afferent sensory fibers from the kidney project to the central nervous system via the dorsal root ganglia (DRG). (C) Schematic illustration of subcapsular injection of tracer into the kidney. (D) Neurons in the SCSMG labeled with CTBPro or CTB‐Alexa488. Confocal: 20×. Scale bar: 20 µm. Laser: 488 nm, 1.0%. (E) High‐resolution imaging of neurons in the SCSMG labeled with CTBPro or CTB‐Alexa488. Confocal: 63×. Scale bar: 10 µm. Laser: 488 nm, 1.0%. (F) Co‐localization of labeled neurons in the SCSMG with tyrosine hydroxylase (TH). Confocal: 63×. Scale bar: 10 µm. Laser: 488 nm, 1.0%. (G) Tracing results of CTBPro or CTB‐Alexa488 in the DRG. Confocal: 20×. Scale bar: 20 µm. Laser: 488 nm, 2.0%. (H) High‐resolution imaging of tracing results in the DRG. Confocal: 63×. Scale bar: 10 µm. Laser: 488 nm, 2.0%. (I) Co‐localization of CTBPro or CTB‐Alexa488 fluorescence signals with TUBB3 in the DRG. Confocal: 63×. Scale bar: 10 µm. Laser: 488 nm, 4.5%. (J) Quantification of CTBPro‐ and CTB‐Alexa488‐labeled neuron numbers in the SCSMG following subcapsular injection. (K) Fluorescence intensity of CTBPro and CTB‐Alexa488 labeling in the SCSMG following subcapsular renal injection. (L) Fluorescence intensity of CTBPro and CTB‐Alexa488 labeling in the DRG following subcapsular renal injection. Each data point represents one mouse (N = 3 per group). Statistical significance was assessed using Student's t‐test.

To validate peripheral neural tracing, we selected the kidney as a target organ. The kidney is extensively innervated by sympathetic nerves, particularly around the afferent arterioles of the glomeruli [23, 24]. In humans, the postganglionic sympathetic neurons innervating the kidney are located in the aorticorenal ganglia. In mice, however, aorticorenal ganglia are absent; instead, ganglia innervating the abdominal viscera are fused into a single complex known as the splanchnic–celiac–superior mesenteric ganglion complex (SCSMG) (Figure 3A) [25]. Previous studies have confirmed the presence of sensory nerve fibers within the kidney, although they are relatively sparse. The cell bodies of these sensory neurons reside in the dorsal root ganglia (DRG), mainly distributed from segments T10 to S2 (Figure 3B) [20].

Following subcapsular injection of CTBPro or CTB‐Alexa488 into the kidney (Figure 3C), a subset of neurons in the SCSMG exhibited fluorescent labeling. Confocal imaging at 20× revealed that CTBPro labeled a greater number of neurons with stronger fluorescence signals, compared to CTB‐Alexa488 (Figure 3D,J,K). High‐resolution 63× confocal imaging of individual neurons further demonstrated that CTBPro produced higher fluorescence intensity and more complete neuronal morphology, allowing clear visualization of axons (Figure 3E). The labeled neurons were further identified as sympathetic neurons (Figure 3F). Similarly, serial sections of DRGs from T10 to S2 were examined, and the region with the highest labeling rate was selected for detailed observation. The distribution of sensory fibers is sparse in the kidney, making it difficult to trace neurons in the DRG. However, CTBPro still successfully labeled neuronal soma in the DRG, while CTB‐Alexa488 failed to do so (Figure 3G–I,L). Thus, CTBPro provides a powerful approach for mapping the DRG‐to‐peripheral organ circuit.

To further verify the reliability of peripheral tracing, we conducted parallel experiments using intraparenchymal renal injection (Figure S4A). No labeling was observed in the DRG with either CTBPro or CTB‐Alexa488. However, in the SCSMG, CTBPro again demonstrated superior tracing ability by labeling a larger number of neurons with stronger fluorescence intensity (Figure S4B–E). Therefore, CTBPro serves as an effective tool for neural tracing of peripheral organs.

### 2.4 Retrograde Tracing With CTBPro in the Cerebrospinal Fluid System

The cerebral ventricles are reportedly innervated by serotonergic neurons originating from the dorsal raphe nucleus (DRN) [26]. A subregion of the DRN, described as the B6 region [27], projects axons into the cerebral ventricles that distribute on the ependymal surface and contact the cerebrospinal fluid (CSF).

**FIGURE 4:**
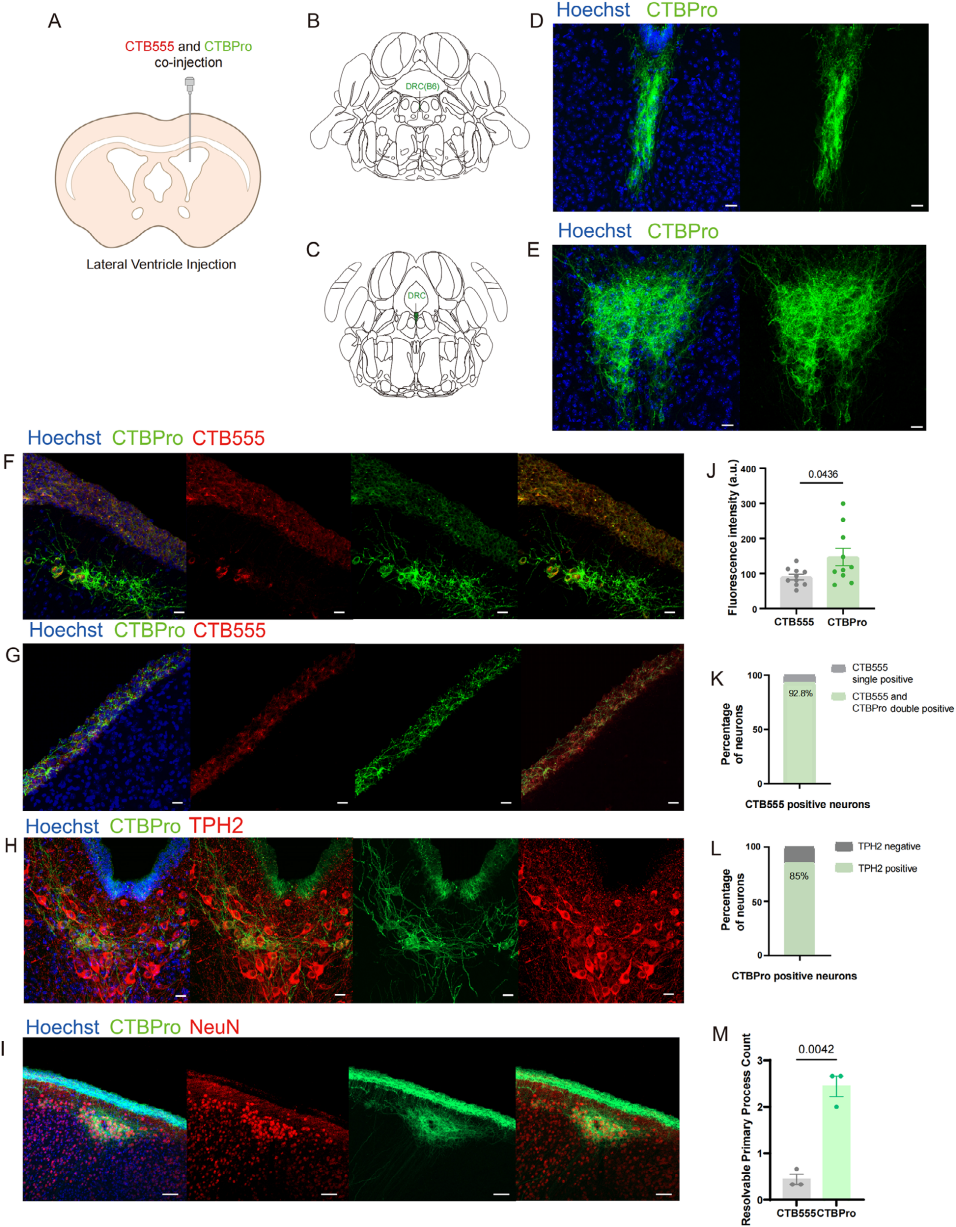
**CTBPro labels CSF‐contacting neurons in the dorsal raphe nucleus (DRN) following lateral ventricle injection**. (A) Schematic illustration of the experiment design of retrograde tracing from lateral ventricle using CTBPro and comparison with CTB‐Alexa555. Equal amount of CTB‐Alexa555 and CTBPro (2.5 µg/µL, 3 µL each) was injected as a mixture into the lateral ventricle. (B) Illustration of the brain region, the caudal part of the dorsal raphe nucleus, which was labeled in red color, corresponding to Figure 4D. (C) Illustration of the brain region, the caudal part of the dorsal raphe nucleus, which was labeled in red color, corresponding to Figure 4E. (D) Labeling pattern of CTBPro in the brain region represented by Figure 4B. Laser: 488 nm, 0.2%. Scale bar: 20 µm. (E) Labeling pattern of CTBPro in the brain region represented by the Figure 4C. Laser: 488 nm, 0.2%. Scale bar: 20 µm. (F) Co‐labeling of CTBPro and CTB‐Alexa555 from sagittal slices. CTBPro achieved better labeling pattern than CTB‐Alexa555 in the soma of neurons. Laser: 488 nm, 0.2%; 555 nm, 2%. Scale bar: 20 µm. (G) Co‐labeling of CTBPro and CTB‐Alexa555 in the supra‐ependymal axonal layer. CTBPro labeled better than CTB‐Alexa555 in the axons of the CSF‐contacting neurons. Laser: 488 nm, 0.2%; 555 nm, 2%. Scale bar: 20 µm. (H) Co‐labeling of CTBPro and TPH2 antibody immunofluorescence staining. Laser: 488 nm, 0.35%; 546 nm, 2.5%. Scale bar: 20 µm. (I) Co‐labeling of CTBPro and NeuN antibody staining. Laser: 488 nm, 0.35%; 546 nm, 1%. Scale bar: 50 µm. (J) Fluorescence intensity comparison between CTBPro and CTB‐Alexa555 (K) Co‐labeling rate of CTB‐Alexa555 and CTBPro signals. (L) Statistics of the co‐labeling rate of TPH2 immunofluorescence signals and the CTBPro positive neurons. (M) Quantification of resolvable primary processes per soma in the DRN.

These are thus known as CSF‐contacting neurons [28]. Using lateral ventricle injections, we further tested whether CTBPro can be used to label this circuitry. Equal volume and concentration of CTBPro and CTB‐Alexa555 were co‐injected into the cerebral ventricles as a mixture. In this way, we could compare the effectiveness of the CTBPro we developed to the commercialized tracers and perform benchmarking of CTBPro (Figure 4A). Brains were harvested 24 h post‐injection. After screening the brain slices, we found labeling of CTBPro in a caudal and dorsal part of the DRN region. The location of the brain region labeled by CTBPro retrograde tracing resembled the DRN region reported by previous studies. The CTBPro signal was exclusively localized to the DRN.

Both soma and the fibers could be labeled by CTBPro, while rich fiber signals could be seen in the DRN (Figure 4B–E). CTBPro labeled nearly the same region as the CTB‐Alexa555, with a high degree of co‐labeling between the two fluorophores (Figure 4K). While CTB‐Alexa555 only labeled parts of the neuronal soma in the DRN, the CTBPro showed the ability to label both the axons and the soma. The efficiency of labeling fibers was higher compared to that of the CTB‐Alexa555 (Figure 4F,G). Consistent with our findings in the VTA‐NAc circuit, we performed a morphological analysis here as well. The results revealed that CTBPro demonstrated far superior morphological characterization compared to CTB‐Alexa555 in this CSF injection pathway (Figure 4M). Furthermore, the overall brightness of labeling the neurons was higher for the CTBPro compared to the CTB‐Alexa555 (Figure 4J).

To show the identity of the cells labeled by CTBPro, we stained for NeuN as a neuronal marker. Axons as well as the somas co‐labeled with the NeuN signals (Figure 4I). Finally, we showed that the neurons labeled by CTBPro were majorly the serotoninergic neurons by co‐staining with TPH2, a typical serotoninergic neuron marker. The CTBPro achieved 85% co‐labeling rate with the marker (Figure 4H–L). This indicated that CTBPro predominantly labeled serotoninergic neurons in the DRN. Taken together, these results indicated that the CTBPro could reliably perform the retrograde tracing in the DRN‐to‐cerebral ventricles circuitry.

### 2.5 Intravascular Delivery of CTBPro Reveals Neuronal Labeling in the PAG

Previous studies have reported that systemic administration of CTB can label preganglionic autonomic neurons as well as a subset of motor neurons located in the brainstem and spinal cord [29, 30]. Building upon these findings, we evaluated the brain‐wide neuronal tracing capability of CTBPro following intravenous administration.

**FIGURE 5:**
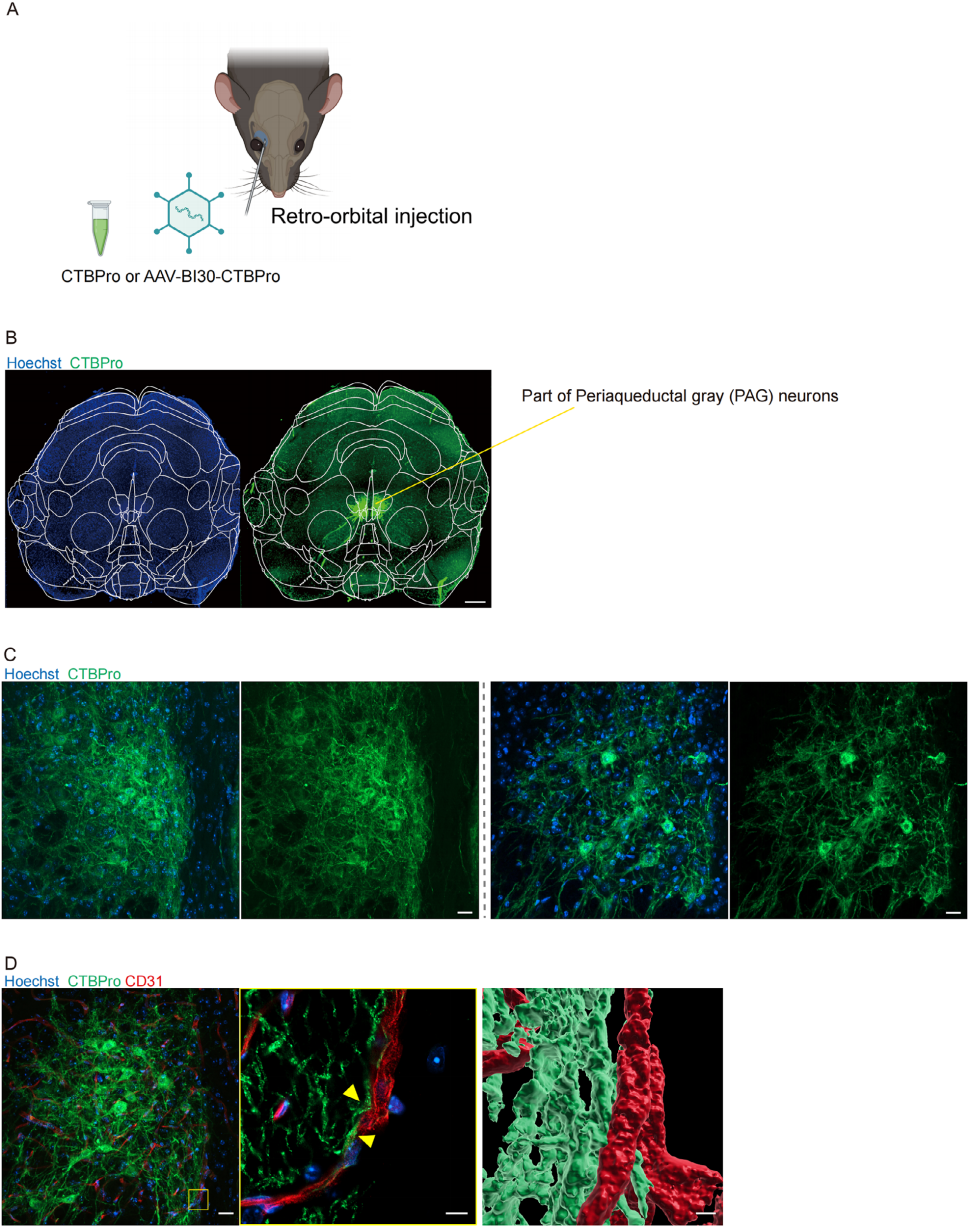
**In vivo neuronal labeling following intravascular injection of CTBPro and AAV‐BI30‐CTBPro**. (A) Schematic of systemic intravascular injection of CTBPro protein or AAV‐BI30‐CTBPro virus. (B) Large‐scale overview showing neuronal labeling in the Periaqueductal Gray (PAG) following systemic CTBPro injection. Scale bar: 500 µm. (C) Detailed view of neuronal labeling in the PAG following intravascular injection of CTBPro protein (left) and AAV‐BI30‐CTBPro virus (right). Scale bar: 20 µm. (D) Visualization of neurovascular contacts labeled by AAV‐BI30‐CTBPro. Left: 20x magnification. Middle: 63x single‐plane magnification. Right: 3D reconstruction using Imaris. Scale bars: 20 µm (left), 10 µm (middle), 2 µm (right).

We first performed daily intravascular injections of purified CTBPro for 7 consecutive days (Figure 5A). Strikingly, while most of the brain showed signals confined to the vasculature, we identified a distinct subset of neurons specifically labeled within the Periaqueductal Gray (PAG) (Figure 5B,C).

To validate this finding and simulate sustained systemic availability, we employed an AAV‐BI30 vector for endothelial‐specific CTBPro expression. After confirming the vector's endothelial specificity (Figure S5A, B), we found that endothelial‐derived CTBPro precisely recapitulated the neuronal labeling pattern, again targeting a specific population exclusively in the PAG (Figure 5C).

To verify the specificity of this labeling, we systematically examined other brain regions and validated the signal's identity. We found that in all other brain regions inspected, the CTBPro signal was confined exclusively to the vascular endothelium, with no neuronal labeling observed. This endothelium‐only pattern was consistent for both protein and AAV‐BI30 delivery (Figure S6A, B). Furthermore, co‐staining with the endothelial marker CD31 confirmed that the vast majority of the CTBPro signal throughout the brain colocalized with the vasculature (Figure S6C).

This evidence highlights that the neuronal labeling is unique to the PAG. 3D reconstruction using Imaris revealed these labeled PAG neurons were in close apposition with vascular endothelial cells (Figure 5D). Finally, to exclude the possibility that this highly localized labeling was due to regional BBB leakage, we administered cadaverine and observed no BBB permeability in this specific area (Figure S7A, B).

These results represent, to our knowledge, the first demonstration of specific CTB‐based neuronal tracing within a distinct brain nucleus following systemic delivery.

## 3 Discussion

Classical retrograde tracers such as cholera toxin subunit B (CTB), Fluoro‐Gold, and retrobeads have been widely used for mapping neural connectivity [31]. Despite their utility, these tools suffer from several limitations, including low fluorescence intensity, limited photostability, and incompatibility with advanced imaging methods such as tissue clearing or electron microscopy. Viral vectors, such as rAAV2‐retro [32] and rabies virus‐based tracers [33], have expanded the experimental repertoire for transsynaptic tracing, but they require long expression times and may introduce immunogenic responses or biosafety concerns.

We have developed CTBPro, a next‐generation CTB‐based retrograde tracer that addresses key limitations of existing tools, offering numerous advantages, including enhanced fluorescence intensity, superior photostability, and broad compatibility with diverse experimental techniques. This novel tracer overcomes many constraints of existing tools, such as low signal intensity and poor morphological resolution. A key improvement is CTBPro's enhanced fluorescence. Compared to conventional CTB‐Alexa488, CTBPro demonstrated a marked increase in intensity, achieving superior performance at a fraction of the molar concentration. Beyond the enhanced fluorescence intensity, the superior image clarity and reduced background of CTBPro likely stem from its structural homogeneity, which contrasts with the stochastic nature of chemical conjugation. Structural analysis of the CTB‐GM1 complex indicates that Lysine 34 and Lysine 91 are positioned on the immediate rim of the GM1‐binding pocket. In commercial chemical conjugates, random attachment of bulky fluorophores to such lysine residues may introduce significant steric hindrance or local conformational distortions [29, 30]. This creates a sub‐population of tracers with compromised binding stability or altered kinetics that contributes to uneven background signals [34]. In contrast, the genetic fusion strategy employed here ensures that five fluorescent protein molecules are precisely conjugated to the five subunits of the CTB pentamer, establishing a defined 1:1 molar ratio. The fluorescent domains are positioned at the C‐terminus via flexible linker peptides, spatially segregating them from the GM1‐binding interface. This design eliminates ‘dark’ competition and steric conflicts, ensuring that every binding event translates into a high‐fidelity fluorescent signal. This allows for clearer, more detailed neuronal labeling without high laser intensities, reducing photodamage.

Furthermore, the successful tracing in three independent circuits highlights its broad applicability. In the CNS, CTBPro results were superior to CTB‐Alexa488 in morphological detail. In the peripheral nervous system, CTBPro outperformed CTB‐Alexa488, producing brighter labeling even in challenging conditions like renal parenchymal injections. In labeling CSF‐contacting neurons, CTBPro showed outstanding performance in visualizing axonal morphologies.

The ability to achieve endogenous expression via AAV vectors is another notable advantage. This facilitates connectivity studies in genetically modified animal models, significantly expanding its utility. This versatility is dramatically highlighted by the novel finding of specific neuronal labeling within the Periaqueductal Gray (PAG) following systemic delivery, both as a purified protein and via AAV‐BI30‐driven endothelial expression. This finding is particularly striking, as CTB is a large protein generally considered BBB‐impermeable. The high specificity of the labeling, which our data suggests is not due to widespread BBB leakage [35], implies that this subpopulation of PAG neurons resides in a unique neurovascular microenvironment. While this study was not designed to dissect this mechanism, this incidental discovery provides a powerful new tool. The specific labeling of these PAG neurons may offer novel insights into the unique structural organization of the neurovascular unit in this region and the intimate nature of its neurovascular interactions.

Additionally, the CTBPro fusion platform can be adapted to integrate other advanced fluorophores, such as myonghong, emiRFP670, or others [21, 22]. This flexibility allows researchers to tailor tracers to specific needs, taking advantage of new developments in fluorescent protein technology. Although CTBPro was successfully expressed, its long‐term in vivo stability and potential cytotoxicity have not been fully assessed. Further studies are needed to evaluate its long‐term safety, especially for longitudinal experiments.

In conclusion, CTBPro offers significant advantages over traditional tracers. Its superior performance in both central and peripheral systems, coupled with its integration with tissue‐clearing and electron microscopy, makes it a powerful tool for modern neuroanatomical studies.

## 4 Experimental Section

### 4.1 Animals

All experimental procedures were in accordance with the National Institutes of Health Guide for the Care and Use of Laboratory Animals in China, and were approved by the Westlake University Institutional Animal Care and Use Committee (IACUC) (24‐013‐JJM). Male and female C57BL/6J mice of at least 8 weeks of age were used for data collection. Animals were housed in a specific pathogen–free (SPF) facility under controlled temperature (22±2°C) and humidity (50±10%) with a 12‐h light/dark cycle and ad libitum access to food and water. Mice were randomly assigned to experimental groups, and all efforts were made to minimize animal suffering.

### 4.2 Protein Expression and Purification

The coding sequence for CTBPro, consisting of a codon‐optimized sequence for CTB (UniProt: Q9UBH6) fused in‐frame to the mBaojin sequence [20], was synthesized by GENEWIZ and confirmed by sequencing. The full‐length coding sequence was cloned into the PCAG expression vector with an N‐terminal signal peptide and a C‐terminal HRV 3C protease cleavage site, followed by a Flag tag. All plasmids for transient expression were confirmed by DNA sequencing. Expi293F cells (Gibco, Thermo Fisher Scientific) were cultured in SMM 293T‐II medium (Sino Biological) at 37°C under 5% CO_2_ in a ZCZY‐CS9 shaker at 150 rpm (Zhichu Instrument). When the cell density reached 2 × 10^6^ cells/mL, 2 mg of the CTBPro plasmid was mixed with 6 mg of linear polyethylenimine (Yeasen) before being added to 1 L of the cell suspension. Transfected cells were harvested after ∼4 days of culture; the supernatant was collected after centrifugation at 4,000 rpm for 10 min for protein purification.

For the purification of CTBPro, 1 L of supernatant was loaded onto Anti‐DYKDDDDK G1 Affinity Resin (GenScript) and washed with 50 mL PBS. On‐column digestion with 3C protease was performed overnight at 4°C. The target protein in the flow‐through was collected the next day, then concentrated to 1 mL using an Amicon Ultra‐4 centrifugal filter (MWCO 50 kDa) (Millipore, Sigma Aldrich) before being injected into a Superdex 200 10/300 GL column (GE Healthcare). SEC was performed in PBS. The peak fractions were collected and concentrated to ∼2.5 mg/mL.

### 4.3 Biochemical Characterization and Deglycosylation Assay

To characterize the apparent molecular weight and electrophoretic mobility of CTBPro, purified protein samples were mixed with SDS‐PAGE loading buffer and subjected to two distinct preparation conditions: heating at 99°C for 15 min or incubation at room temperature (unboiled) prior to loading. Both samples were resolved on SDS‐PAGE gels to compare their migration patterns.

To verify the presence of glycosylation, enzymatic deglycosylation was performed using PNGase F (Cat. No. P0704S, New England Biolabs) following the manufacturer's instructions. Briefly, the protein was denatured and incubated with PNGase F to remove N‐linked glycans. The reaction products were subsequently mixed with loading buffer, heated at 99°C for 15 min, and analyzed by SDS‐PAGE to visualize the shift in molecular weight.

### 4.4 High‐Resolution Mass Spectrometry and Protein Identification

Mass spectrometry analyses were performed at the Mass Spectrometry & Metabolomics Core Facility (MASCOF) of Westlake University.

*Intact Mass Analysis (Molecular Weight Determination)*: To determine the precise molecular weight, protein samples (both native and deglycosylated) were analyzed by High‐Resolution Intact Protein LC‐MS. The raw mass spectra were acquired, and the zero‐charge molecular masses were determined using deconvolution algorithms to reveal the precise molecular weight of the protein backbone.

*Peptide Mapping and Protein Identification*: To confirm the protein sequence and identify post‐translational modifications, the deglycosylated CTBPro band was excised from the SDS‐PAGE gel and subjected to in‐gel tryptic digestion. The resulting peptide mixture was analyzed by Liquid Chromatography‐Tandem Mass Spectrometry (LC‐MS/MS). The acquired MS/MS spectra were searched against the theoretical CTBPro sequence to validate sequence coverage and identify glycosylation sites.

### 4.5 CTBPro Structure Analysis

The structural integrity of CTBPro was assessed using AlphaFold3 [36]. To focus on the rigid components critical for binding, the flexible alkane chain of GM1 was omitted. Structural comparisons were performed by aligning these predicted models with the experimentally determined crystal structure of CTB (PDB ID: 6HMY), yielding an RMSD of 0.194 Å, indicating minimal structural distortion in CTBPro upon mBaojin fusion.

Hydrogen bond interactions between GM1 and native CTB/CTBPro were analyzed using PLIP [37]. We performed docking of GM1 with native CTB and CTBPro using AutoDock Vina [38, 39].

### 4.6 Brain Stereotactic Injection

Animals were anesthetized with isoflurane at 3% concentration for 5 min for induction. During surgery and CTB‐Alexa488 (C22841, Invitrogen)/CTBPro injections, animals were kept anesthetized with isoflurane at 1.5% concentration. A heating pad (37 ± 0.5°C) was used throughout the procedure. Injections were conducted using a stereotaxic injection system (RWD, 71001‐S). A total volume of 100 nL was delivered to the NAc (AP: +1.50 mm, ML: ±0.73 mm, DV: −4.23 mm from bregma) at a flow rate of 1 nL/s. All injected solutions (CTB‐Alexa488/CTBPro) were used at a concentration of 2.5 µg/µL.

### 4.7 Kidney Subcapsular Injection

Mice were anesthetized as described for stereotaxic surgery. The mouse was placed in a prone position on the surgical platform, and the limbs were gently secured if necessary. The fur over the left dorsal flank region was shaved, and a small skin incision was made near the spine to expose the left kidney. A 0.3‐mL insulin syringe was used to gently lift the renal capsule, and the needle was inserted almost parallel to the kidney surface until the tip was fully positioned beneath the capsule. Subsequently, 10 µL of CTB‐Alexa488 or CTBPro was injected into one kidney. After confirming diffusion and infiltration of the injected solution under the capsule, the needle was carefully withdrawn. The muscle and skin layers were sutured, and the mouse was allowed to recover under standard conditions. Tissues were collected 5 days after injection.

### 4.8 Kidney Intraparenchymal Injection

The preparation of mice was identical to that described for subcapsular injection. Intraparenchymal renal injections were performed using a microinjection pump. A custom‐made glass micropipette was connected to the pump and filled with CTB‐Alexa488 or CTBPro. The micropipette was slowly lowered until it penetrated the renal capsule and entered the parenchyma, with the depth controlled at 0.1–0.2 cm. Four injection sites were selected on the same kidney, and 1 µL of tracer was injected per site at a constant rate of 0.1 µL/min. After each injection, the micropipette was left in place for 2–5 min to allow diffusion of the tracer and minimize reflux. The micropipette was then slowly withdrawn to avoid hemorrhage caused by sudden pressure release, and a small amount of mineral oil was applied to the injection site to seal it.

### 4.9 Histology

Mice were anesthetized with 1% sodium pentobarbital and perfused transcardially with pre‐cooled 1x phosphate‐buffered saline (PBS) followed by 4% paraformaldehyde (PFA). Brains were post‐fixed in 4% PFA overnight, embedded in 5% agarose, and sectioned coronally at 40–50 µm thickness using a vibrating microtome (Leica VT1200S).

For ganglion collection, animals were perfused with PBS, and the dissected ganglia were immediately fixed in 4% PFA for 4–6 h. The tissues were then cryoprotected in PBS containing 30% sucrose, embedded in OCT compound, and cryosectioned at 20 µm thickness using a cryostat (Epredia CryoStar NX70).

### 4.10 Immunofluorescence and Confocal Imaging

All tissue sections were blocked and permeabilized for 2 h in PBS containing 5% normal donkey serum (NDS; 017‐000‐121, Jackson ImmunoResearch Inc.) or normal goat serum (NGS; 005‐000‐121, Jackson ImmunoResearch Inc.) and 1% Triton X‐100 (A110694‐0500, Sangon Biotech, Shanghai, China). Sections were then incubated with primary antibodies, including anti‐NeuN (1:1000, MAB377, Sigma‐Aldrich), anti‐TPH2 (1:500, OB‐PRT105, Oasis Biofarm), anti‐TH (1:300, OPA1‐04050, Invitrogen), and anti‐TUBB3 (1:500, OB‐PRB062, Oasis Biofarm). After overnight incubation with primary antibodies at 4°C, sections were washed and incubated with appropriate secondary antibodies (donkey anti‐mouse, Yeasen; donkey anti‐rat, Jackson ImmunoResearch; goat anti‐rabbit, Thermo Fisher) for 2 h at room temperature. Sections were counterstained with DAPI (1 µg/mL) and mounted with antifade mounting medium (Thermo Fisher) before imaging.

Mouse sample imaging was performed using a Zeiss LSM 900 laser scanning microscope with 20× and 63× objectives or Zeiss Axioscan7 with 20× objective. Image processing was carried out using ZEN Microscopy Software (Zeiss) or Imaris 10.0 (Bitplane). In some cases, fluorescence data were further reconstructed in Imaris 10.0. For quantitative comparison, laser power, gain, and offset were kept constant across samples. Fluorescence intensity and cell counting were quantified using ZEN or ImageJ.

### 4.11 Lateral Ventricle Injection

Mice were anesthetized as described for stereotaxic surgery. The scalp was shaved, depilated, and sterilized with povidone‐iodine. A midline incision exposed the skull, and a small burr hole (0.5 mm) was drilled over the right lateral ventricle (coordinates from bregma: AP −0.27 mm, ML +1.04 mm, DV −2.08 mm). A 25‐µL Hamilton syringe (1701 RN; 26‐gauge, point style 4) on a micro‐infusion pump (KD Scientific) was lowered to the target site at 0.2 mm/min. A total volume of 6 µL, consisting of 3 µL CTBPro (0.25 mg/mL in aCSF, pH 7.4, 0.22‐µm filtered) and 3 µL CTB‐Alexa555 (0.25 mg/mL in aCSF, pH 7.4, 0.22‐µm filtered), was delivered at 0.2 µL/min. The needle was left in place for 10 min post‐injection before slow withdrawal (0.2 mm/min). The skin was sutured (6‐0 silk), and meloxicam (0.1 mg/kg, s.c.) was administered for analgesia. Animals were monitored until fully ambulatory.

### 4.12 iDISCO Optical Tissue Clearing and Imaging

iDISCO permits whole‐mount immunolabeling with volume imaging of large cleared samples ranging from perinatal mouse embryos to adult organs, such as brains or kidneys [40]. Brain samples were washed three times (60 min each) in PBS at RT on an orbital shaker (Benchmark, Roto‐Bot), then incubated in 30% sucrose at RT. Dehydration was performed at RT in a graded methanol series (20%, 40%, 60%, 80%, 100% v/v in H_2_O, 1 h each). Samples were incubated in 100% methanol overnight at 4°C. Endogenous pigments were bleached by incubation in 5% H_2_O_2_ (Ren‐Bang Medicine) in 100% methanol for 24 h at 4°C, protected from light. Samples were rehydrated (80%, 60%, 40%, 20% methanol, 1 h each) and washed twice (30 min each) in PBS. Samples were dehydrated again, followed by two 2‐hour incubations in 66% dichloromethane (DCM, Energy Chemical) / 33% methanol at RT. After three 2‐hour washes in 100% DCM, tissues were cleared by immersion in BB‐BED solution (47% Benzyl benzoate [Sigma], 48% BED468 [Sigma], 5% Quadol [Sigma]) overnight. Imaging was performed using a Zeiss Z7 light‐sheet microscope.

### 4.13 Intravenous CTBPro Injection

Mice were anesthetized as described for stereotaxic surgery. For systemic injections, 50 µL of CTBPro (2.5 µg/µL) was diluted to 150 µL with sterile physiological saline (0.22 µm‐filtered). This solution was administered via retro‐orbital injection daily for seven consecutive days, alternating orbits.

### 4.14 Virus Injection

For AAV‐BI30‐Cre and AAV‐BI30‐CTBPro injections, each mouse received 1 × 10^1^ ^1^ vector genomes (vg) via retro‐orbital injection, a titer selected based on published reports [41, 42]. Brain tissues were harvested four weeks post‐injection.

### 4.15 Fluorescence Measurements

Photoluminescence (PL) spectra were recorded on a Photoluminescence Spectrometer (Edinburgh Instrument FLS1000). Prior to measurement, stock samples (2.5 µg/µL) were diluted 1000‐fold with ddH_2_O. Fluorescence intensity was evaluated at various excitation wavelengths.

### 4.16 Quantification and Statistics

All fluorescence intensity data of in vivo experiment analyses were performed using ZEN 3.8 software (Zeiss). For statistical analysis of tissue sections, three representative slices were selected from each mouse. On each slice, fluorescence intensity was measured from five distinct regions of interest (ROIs), and these values were averaged to obtain a representative value for each animal. To quantify the morphological fidelity of each tracer, the average number of resolvable primary processes extending from labeled somata was manually counted. A strict criterion was established: a “resolvable primary process” was defined as a continuous neurite extending more than 3 µm from the soma border, with a fluorescence intensity at least twofold greater than the local background. Statistical analyses were performed using the R statistical software or GraphPad Prism 10.5.0. The data were presented as the mean value ± standard error of the mean (SEM). Depending on the data distribution and variance, different tests were employed for two‐group comparisons: Student's t‐test, Welch's t‐test, or the Wilcoxon rank sum test.

## Conflicts of Interest

A patent application related to the CTBPro technology described in this manuscript has been filed by Westlake University with J.‐M.J., X.Q., Y.W., Y.Z., L.Y., Y.L., and H.X. listed as inventors. The remaining authors declare no competing interests.

[Correction added on 01 May 2026 after first online publication: Patent statement is added.]

## Supporting information




**Supporting File 1**: advs75424‐sup‐0001‐SuppMat.docx.

**Supporting File 2**: advs75424‐sup‐0002‐FigureS1‐S7.zip.

**Supporting File 3**: advs75424‐sup‐0003‐TableS1‐S3.zip.

## Data Availability

The data that support the findings of this study are available from the corresponding author upon reasonable request.
