# Supplementary material for: CTBPro: A Next‐Generation Cholera Toxin Subunit B‐Based Neuroanatomical Tracer With Superior Brightness, Stability, and Sensitivity for Enhanced Neural Circuit Mapping
Source: Adv Sci (Weinh). 2026 Apr 22;13(40):e22249. doi: 10.1002/advs.202522249 (PMC13335673; doi:10.1002/advs.202522249)
Supplement: Supplementary file 1 — Supporting File 1: advs75424‐sup‐0001‐SuppMat.docx. [file ADVS-13-e22249-s002.docx]

Supporting Information

CTBPro: A Next-Generation Cholera Toxin Subunit B-Based Neuroanatomical Tracer with Superior Brightness, Stability, and Sensitivity for Enhanced Neural Circuit Mapping

Xinghua Quan^1,3,#^, Yude Lou^1,3,#^, Huiqi Xie^1,3,#^, Yifei Wang^5,#^, Yanzhe Zhang^2,3^, Yiran Ge^1^, Linhe Yang^2,6^, Xiaoxuan Zhang^1^, Qingmiao Zhou^5^, Huaizong Shen^5^, Longxing Cao^2,3^, Xin Jin^2,6^, Jie-Min Jia^1,2,3,4,*^

**Figure S1. Comprehensive biochemical characterization and sequence validation of CTBPro.** (A) SDS-PAGE analysis of thermal stability and glycosylation status. (Left Panel) Loading and Boiling Assay: Purified CTBPro was loaded at increasing amounts (0.625, 1.25, and 2.5 µg). Samples were either maintained at room temperature (Unboiled, -) or heated at 99℃ for 15 min (Boiled, +). Boiling facilitates the resolution of the monomeric species, while unboiled samples exhibit distinct electrophoretic mobility. (Right Panel) Deglycosylation Assay: Comparison of native (Lane 1) and PNGase F-treated (Lane 2) CTBPro (both boiled). The native protein migrates as a diffuse smear (~45–55 kDa) due to glycosylation, whereas enzymatic deglycosylation collapses the smear into a single, sharp band at approximately 40 kDa, consistent with the theoretical backbone size. (B) High-resolution intact mass spectrometry. (Left) Raw spectrum of native CTBPro showing a heterogeneous envelope characteristic of complex glycosylation. (Right) Deconvoluted spectrum of the deglycosylated backbone. The primary species has a measured mass of 39,639.0 Da, matching the theoretical mass (39,644.0 Da) with a deviation of < 5 Da. (C) Peptide mapping and sequence validation. LC-MS/MS analysis of the deglycosylated sample achieved ~97% sequence coverage. Identified peptides (highlighted in red) confirm the integrity of the full-length fusion protein. (D) Analysis of oligomeric state by Size-Exclusion Chromatography (SEC). CTBPro elutes as a single symmetrical peak at ~11.5 mL. The elution position, calibrated against standard markers (Thyroglobulin, Ferritin, Aldolase, Ovalbumin; dotted lines), corresponds to an apparent molecular weight of ~240 kDa, confirming the assembly of a stable pentamer.

**Figure S2. Compatibility of CTBPro labeling with tissue clearing.** (A) Axonal signals labeled by CTBPro in the ipsilateral cortex (injection side) after iDISCO (left) and somatic signals in the contralateral cortex (right). Scale bar: 100 µm.

**Figure S3. In silico docking analysis of CTBPro to the GM3 ganglioside.** (A) Predicted docking pose of CTBPro with the GM3 ganglioside, showing lower affinity compared to GM1.

**Figure S4. Comparison of renal neural tracing between CTBPro and CTB-Alexa488 by intraparenchymal injection.** (A) Intraparenchymal renal injection. (B) Neurons in the SCSMG labeled with CTBPro or CTB-Alexa488. Confocal: 20×. Scale bar: 20 µm. Laser: 488 nm, 2.0%. (C) Quantification of CTBPro- and CTB-Alexa488-labeled neuron numbers and fluorescence intensity in the SCSMG following intraparenchymal injection. (D) High-resolution imaging of neurons in the SCSMG labeled with CTBPro or CTB-Alexa488. Confocal: 63×. Scale bar: 10 µm. Laser: 488 nm, 2.0%. (E) Co-localization of labeled neurons in the SCSMG with tyrosine hydroxylase (TH). Confocal: 63×. Scale bar: 10 µm. Laser: 488 nm, 2.0%. Each data point represents one mouse (N = 3 per group). Statistical significance was assessed using Student's t-test.

**Figure S5. Specificity of AAV-BI30.** (A) Representative images showing endothelial-specific expression of AAV-BI30-Cre in Ai14 reporter mice. Scale bar: 500 µm. (B) Quantification of the colocalization rate between the AAV-BI30 signal and endothelial markers (CD31), analyzed using the JAcoP plugin for ImageJ.

**Figure S6. Brain-wide specificity and endothelial colocalization of systemically delivered CTBPro.** (A) Representative images showing CTBPro signal in different brain regions following intravenous protein injection. Scale bar: 500 µm. (B) Representative images showing CTBPro signal in different brain regions following AAV-BI30-CTBPro injection, similarly demonstrating endothelium-restricted labeling. Scale bar: 500 µm. (C) Representative image and quantification of the colocalization between the CTBPro signal (green) and the endothelial marker CD31 (red). Scale bar: 50 µm.

**Figure S7. BBB leakage detection in the PAG region.** (A) Positive control of BBB leakage detection by cadaverine-647 injection in a stroke model. Scale bar: 50 µm. (B) Cadaverine-647 injection result in the PAG region, showing no detectable leakage. Scale bar: 50 µm.

**Table S1. Complete nucleotide and amino acid sequences of the CTBPro construct.**

**Table S2. Peptide mapping and sequence coverage analysis of deglycosylated CTBPro by LC-MS/MS.** The complete cleaved CTBPro sequence used for MS/MS database searching includes the C-terminal remnant GTGSLEVLFQ.

**Table S3. Multi scan data of CTB-Alexa488 and CTBPro.**

| Parameter | CTBPro | CTB-Alexa488 |
| --- | --- | --- |
| Labels | EmScan-ctbpro | EmScan-ctb488 |
| Type | Emission Scan | Emission Scan |
| Comment |  |  |
| Start | 491.00 | 491.00 |
| Stop | 600.00 | 600.00 |
| Step | 1.00 | 1.00 |
| Fixed/Offset | 488.00 | 488.00 |
| Xaxis | Wavelength | Wavelength |
| Yaxis | Counts | Counts |
| Scan Corr. by File | True | True |
| Corr. by Ref. Det. | True | True |
| Fixed/Offset Corr. by File | False | False |
| Repeats | 1 | 1 |
| Dwell Time | 0.30 | 0.30 |
| Lamp | Xenon | Xenon |
| Temp | 0.00 | 0.00 |
| Scan Polariser | None | None |
| Scan Slit | 2.0000091 | 2.0000091 |
| Fixed/Offset Polariser | None | None |
| Fixed/Offset Slit | 1.9999765 | 1.9999765 |
| Detector | PMT980 | PMT980 |
| 492.00 | 9.91968828E+4 | 1.87515098E+4 |
| 493.00 | 1.27482367E+5 | 2.19999668E+4 |
| 494.00 | 1.64764313E+5 | 2.68563086E+4 |
| 495.00 | 2.07902172E+5 | 3.26455156E+4 |
| 496.00 | 2.60028531E+5 | 3.75821523E+4 |
| 497.00 | 3.16085781E+5 | 4.68389023E+4 |
| 498.00 | 3.80298719E+5 | 5.41263633E+4 |
| 499.00 | 4.51080875E+5 | 6.34186641E+4 |
| 500.00 | 5.20315188E+5 | 7.30908047E+4 |
| 501.00 | 5.91369688E+5 | 8.58449609E+4 |
| 502.00 | 6.46949000E+5 | 9.70090625E+4 |
| 503.00 | 6.94194000E+5 | 1.09040297E+5 |
| 504.00 | 7.21852125E+5 | 1.22121609E+5 |
| 505.00 | 7.37804938E+5 | 1.35610844E+5 |
| 506.00 | 7.39118500E+5 | 1.47559750E+5 |
| 507.00 | 7.22494500E+5 | 1.64245359E+5 |
| 508.00 | 6.94243250E+5 | 1.77516813E+5 |
| 509.00 | 6.60100188E+5 | 1.88995203E+5 |
| 510.00 | 6.13805625E+5 | 2.00662094E+5 |
| 511.00 | 5.77390063E+5 | 2.15281266E+5 |
| 512.00 | 5.25487625E+5 | 2.25170938E+5 |
| 513.00 | 4.92468719E+5 | 2.33496688E+5 |
| 514.00 | 4.52578250E+5 | 2.41311313E+5 |
| 515.00 | 4.20763406E+5 | 2.50510078E+5 |
| 516.00 | 3.91918188E+5 | 2.53715031E+5 |
| 517.00 | 3.67114219E+5 | 2.59487344E+5 |
| 518.00 | 3.48534406E+5 | 2.64794406E+5 |
| 519.00 | 3.29821656E+5 | 2.68384156E+5 |
| 520.00 | 3.10042250E+5 | 2.64511750E+5 |
| 521.00 | 2.97638625E+5 | 2.65133500E+5 |
| 522.00 | 2.88435344E+5 | 2.65740594E+5 |
| 523.00 | 2.75137281E+5 | 2.62415875E+5 |
| 524.00 | 2.58912281E+5 | 2.57415297E+5 |
| 525.00 | 2.49867078E+5 | 2.51527641E+5 |
| 526.00 | 2.43928563E+5 | 2.46908172E+5 |
| 527.00 | 2.34752719E+5 | 2.41436344E+5 |
| 528.00 | 2.27815703E+5 | 2.35021703E+5 |
| 529.00 | 2.23478047E+5 | 2.25229609E+5 |
| 530.00 | 2.16037313E+5 | 2.20327188E+5 |
| 531.00 | 2.09119703E+5 | 2.11070422E+5 |
| 532.00 | 2.07264141E+5 | 2.04672641E+5 |
| 533.00 | 2.05283156E+5 | 1.99306984E+5 |
| 534.00 | 2.05601078E+5 | 1.93440031E+5 |
| 535.00 | 2.01479891E+5 | 1.85429938E+5 |
| 536.00 | 1.99934297E+5 | 1.81023156E+5 |
| 537.00 | 1.98233672E+5 | 1.73449250E+5 |
| 538.00 | 1.96270109E+5 | 1.65079469E+5 |
| 539.00 | 1.94141313E+5 | 1.57410359E+5 |
| 540.00 | 1.93630875E+5 | 1.51507953E+5 |
| 541.00 | 1.89930828E+5 | 1.46603469E+5 |
| 542.00 | 1.85335281E+5 | 1.44160641E+5 |
| 543.00 | 1.80698094E+5 | 1.40657844E+5 |
| 544.00 | 1.77905594E+5 | 1.32462266E+5 |
| 545.00 | 1.68761547E+5 | 1.31220563E+5 |
| 546.00 | 1.65435250E+5 | 1.23848484E+5 |
| 547.00 | 1.55562406E+5 | 1.20107773E+5 |
| 548.00 | 1.51239563E+5 | 1.16739406E+5 |
| 549.00 | 1.40541813E+5 | 1.13043695E+5 |
| 550.00 | 1.33162078E+5 | 1.08789594E+5 |
| 551.00 | 1.26789344E+5 | 1.04124859E+5 |
| 552.00 | 1.19219164E+5 | 1.01366930E+5 |
| 553.00 | 1.11278109E+5 | 1.01988195E+5 |
| 554.00 | 1.04938344E+5 | 9.49587500E+4 |
| 555.00 | 1.00623242E+5 | 9.46462109E+4 |
| 556.00 | 9.69523828E+4 | 9.43634766E+4 |
| 557.00 | 8.96507266E+4 | 8.92791406E+4 |
| 558.00 | 8.70916250E+4 | 8.73309453E+4 |
| 559.00 | 8.03863594E+4 | 8.57573281E+4 |
| 560.00 | 7.73435156E+4 | 8.19239063E+4 |
| 561.00 | 7.51123984E+4 | 7.99256641E+4 |
| 562.00 | 6.96324141E+4 | 7.78357813E+4 |
| 563.00 | 6.86376797E+4 | 7.47953359E+4 |
| 564.00 | 6.36023789E+4 | 7.41511563E+4 |
| 565.00 | 6.12926484E+4 | 7.05261641E+4 |
| 566.00 | 5.76939922E+4 | 6.93969219E+4 |
| 567.00 | 5.52249102E+4 | 6.67457891E+4 |
| 568.00 | 5.43598203E+4 | 6.26460898E+4 |
| 569.00 | 5.07048750E+4 | 6.38504023E+4 |
| 570.00 | 4.87638945E+4 | 5.90966328E+4 |
| 571.00 | 4.53503242E+4 | 5.81569766E+4 |
| 572.00 | 4.42958711E+4 | 5.53464023E+4 |
| 573.00 | 4.22180313E+4 | 5.50062617E+4 |
| 574.00 | 4.34822305E+4 | 5.25448320E+4 |
| 575.00 | 4.01967500E+4 | 4.92175820E+4 |
| 576.00 | 3.87860938E+4 | 4.85278164E+4 |
| 577.00 | 3.82403516E+4 | 4.54307617E+4 |
| 578.00 | 3.68955352E+4 | 4.51910195E+4 |
| 579.00 | 3.70429414E+4 | 4.29357070E+4 |
| 580.00 | 3.51195078E+4 | 4.11573242E+4 |
| 581.00 | 3.40465898E+4 | 3.99442266E+4 |
| 582.00 | 3.36462109E+4 | 3.92094414E+4 |
| 583.00 | 3.13853242E+4 | 3.71765820E+4 |
| 584.00 | 2.92549551E+4 | 3.60876992E+4 |
| 585.00 | 2.99659727E+4 | 3.40503477E+4 |
| 586.00 | 2.87633574E+4 | 3.32994883E+4 |
| 587.00 | 2.75892246E+4 | 3.13762148E+4 |
| 588.00 | 2.70735977E+4 | 3.02544492E+4 |
| 589.00 | 2.60437500E+4 | 2.77992090E+4 |
| 590.00 | 2.55364941E+4 | 2.75032676E+4 |
| 591.00 | 2.37311934E+4 | 2.72242207E+4 |
| 592.00 | 2.23479180E+4 | 2.43546094E+4 |
| 593.00 | 2.10336348E+4 | 2.43299824E+4 |
| 594.00 | 2.07342949E+4 | 2.36724375E+4 |
| 595.00 | 2.11199727E+4 | 2.25190840E+4 |
| 596.00 | 1.95166680E+4 | 2.19782090E+4 |
| 597.00 | 1.81091055E+4 | 2.16355820E+4 |
| 598.00 | 1.88487598E+4 | 2.07369473E+4 |
| 599.00 | 1.81938418E+4 | 1.87159961E+4 |
| 600.00 | 1.57248418E+4 | 1.89037266E+4 |
