# Supplementary material for: CTBPro: A Next‐Generation Cholera Toxin Subunit B‐Based Neuroanatomical Tracer With Superior Brightness, Stability, and Sensitivity for Enhanced Neural Circuit Mapping
Source: Adv Sci (Weinh). 2026 Apr 22:e22249. Online ahead of print. doi: 10.1002/advs.202522249 (PMC13335673; doi:10.1002/advs.202522249)
Supplement: Supplementary file 1 [file ADVS-9999-e22249-s001.zip › Figure S1-2nd revised.pdf]

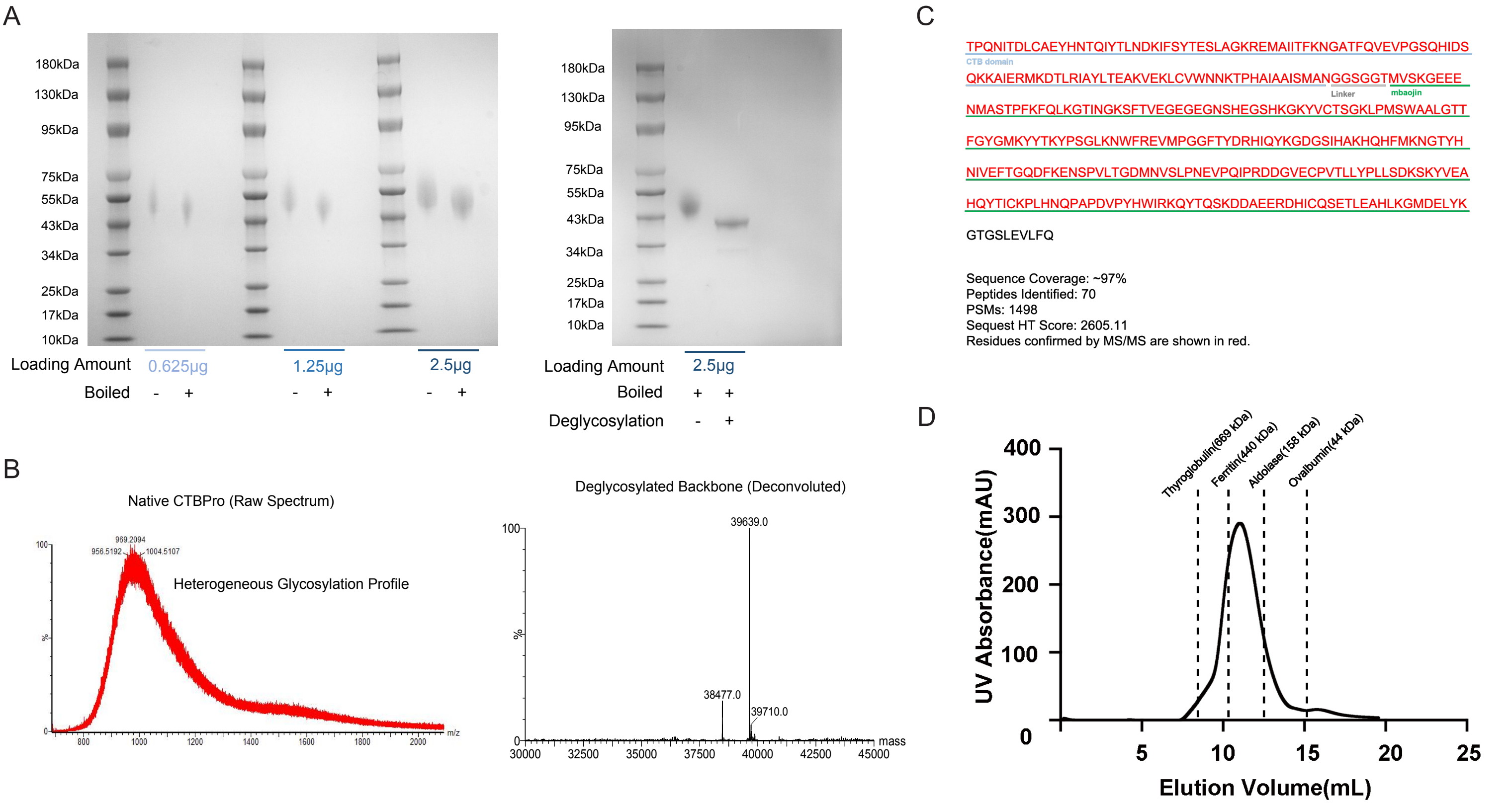
A
180kDa
130kDa
95kDa
75kDa
55kDa
43kDa
34kDa
25kDa
17kDa
10kDa
Loading Amount
0.625μg
1.25μg
2.5μg
Boiled - + - + - +
Loading Amount 2.5μg
Boiled + +
Deglycosylation - +
B
Native CTBPro (Raw Spectrum)
969.2094
956.5192
1004.5107
Heterogeneous Glycosylation Profile
m/z
Deglycosylated Backbone (Deconvoluted)
39639.0
38477.0
39710.0
mass
C
TPQNITDLCAEYHNTQIYTLNDKIFSYTESLAGKREMAIITFKNGATFQVEVPGSQHIDS
CTB domain
QKKAIERMKDTLRIAYLTEAKVEKLCVWNNKTPHAIAAISMANGGSGGTMVSKGEEE
Linker
mbaojin
NMASTPFKFQLKGTINGKSFTVEGEGEGNSHEGSHKGKYVCTSGKLPMSWAALGTT
FGYGMKYYTKYPSGLKNWFREVMPGGFTYDRHIQYKGDGSIHAKHQHFMKNGTYH
NIVEFTGQDFKENSPVLTGDMNVSLPNEVPQIPRDDGVECPVTLLYPLLSDKSKYVEA
HQYTICKPLHNQPAPDVPYHWIRKQYTQSKDDAEERDHICQSETLEAHLKGMDELYK
GTGSLEVLFQ
Sequence Coverage: ~97%
Peptides Identified: 70
PSMs: 1498
Sequest HT Score: 2605.11
Residues confirmed by MS/MS are shown in red.
D
Thyroglobulin(669 kDa)
Ferritin(440 kDa)
Aldolase(158 kDa)
Ovalbumin(44 kDa)
UV Absorbance(mAU)
Elution Volume(mL)
