# Supplementary material for: CTBPro: A Next‐Generation Cholera Toxin Subunit B‐Based Neuroanatomical Tracer With Superior Brightness, Stability, and Sensitivity for Enhanced Neural Circuit Mapping
Source: Adv Sci (Weinh). 2026 Apr 22:e22249. Online ahead of print. doi: 10.1002/advs.202522249 (PMC13335673; doi:10.1002/advs.202522249)
Supplement: Supplementary file 1 [file ADVS-9999-e22249-s001.zip › Figure S4-revised.pdf]

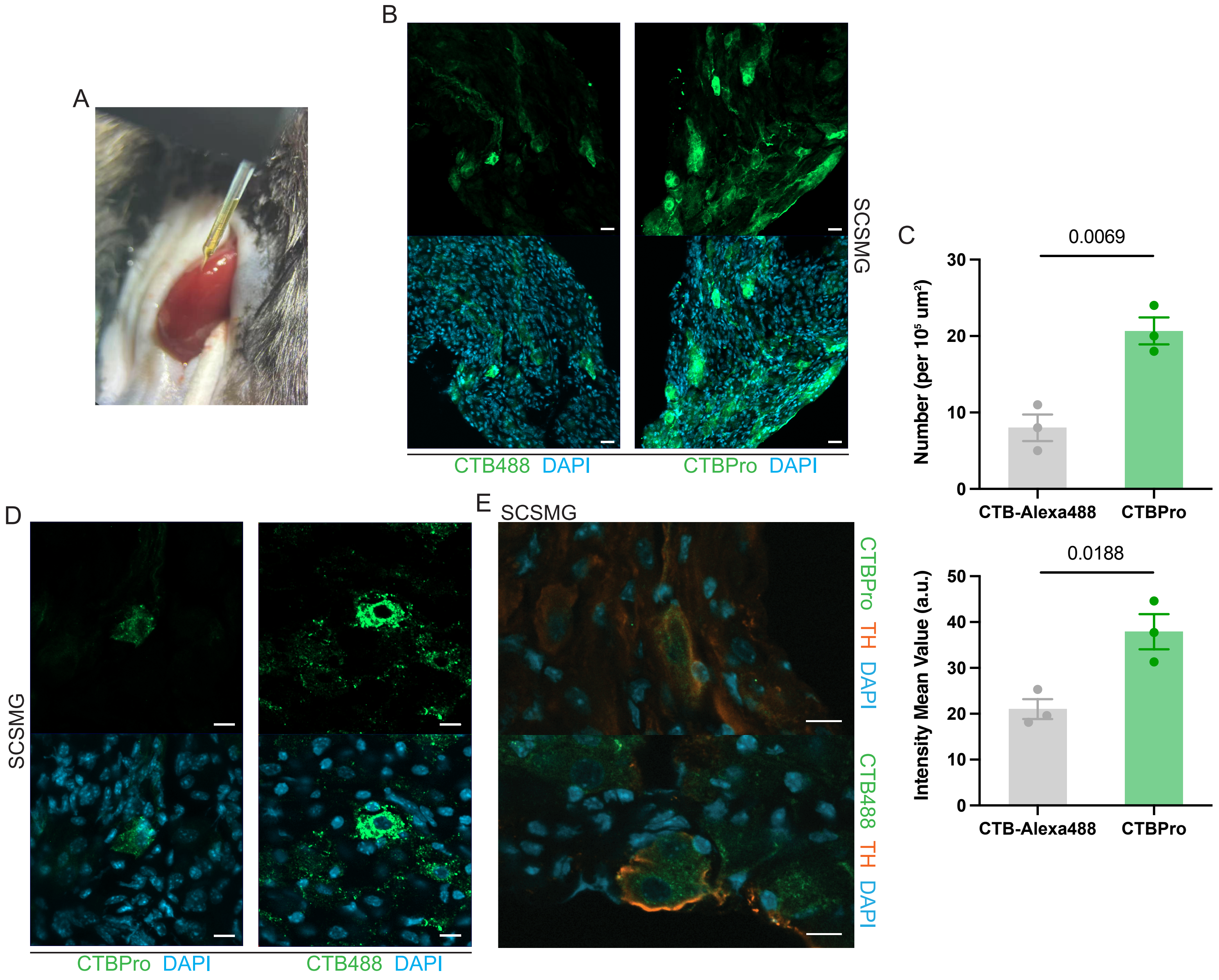
A
B
SCSMG
CTB488 DAPI
CTBPro DAPI
C
0.0069
Number (per 10^5 um^2)
0
10
20
30
CTB-Alexa488
CTBPro
0.0188
Intensity Mean Value (a.u.)
0
10
20
30
40
50
CTB-Alexa488
CTBPro
D
SCSMG
CTBPro DAPI
CTB488 DAPI
E
SCSMG
CTBPro TH DAPI
CTB488 TH DAPI
