# Supplementary material for: CTBPro: A Next‐Generation Cholera Toxin Subunit B‐Based Neuroanatomical Tracer With Superior Brightness, Stability, and Sensitivity for Enhanced Neural Circuit Mapping
Source: Adv Sci (Weinh). 2026 Apr 22:e22249. Online ahead of print. doi: 10.1002/advs.202522249 (PMC13335673; doi:10.1002/advs.202522249)

A

Hoechst Cadaverine647

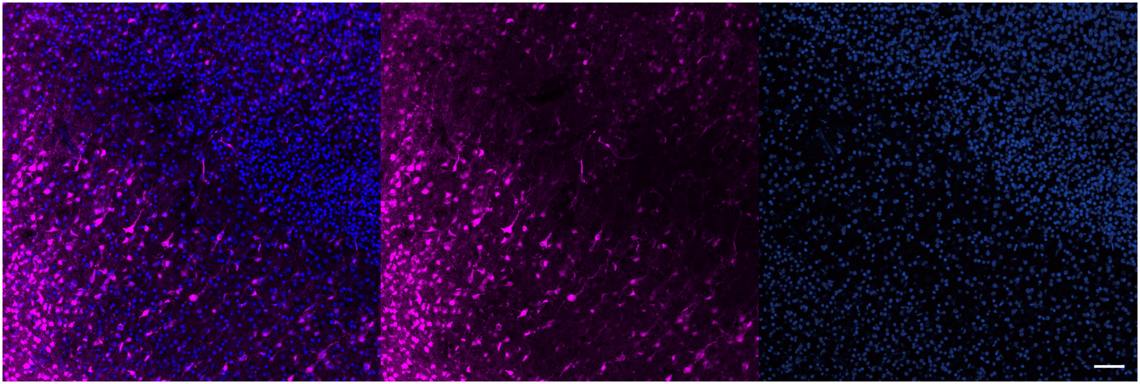

B

Hoechst Cadaverine647 NEUN

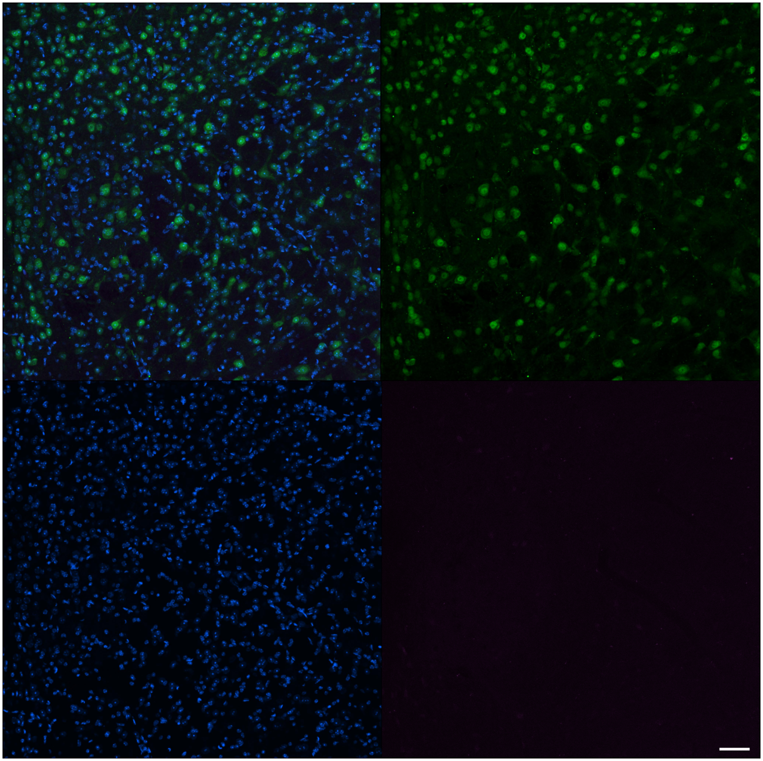

Supplement: Supplementary file 1 [file ADVS-9999-e22249-s001.zip › Figure S7-revised.pdf]
